# Relationship between Serum Cortisol, Dehydroepiandrosterone Sulfate (DHEAS) Levels, and Natural Killer Cell Activity: A Cross-Sectional Study

**DOI:** 10.3390/jcm12124027

**Published:** 2023-06-13

**Authors:** Eunkyung Suh, A-Ra Cho, Ji-Hee Haam, Minchan Gil, Yun-Kyong Lee, Young-Sang Kim

**Affiliations:** 1 Chaum Life Center, CHA University, Seoul 06062, Republic of Korea; sherby@chamc.co.kr; 2 Department of Family Medicine, Gangnam Severance Hospital, Yonsei University College of Medicine, Seoul 06273, Republic of Korea; ara1713@yuhs.ac; 3 Department of Family Medicine, CHA Bundang Medical Center, CHA University, Seongnam 13496, Republic of Korea; hamjhi@chamc.co.kr; 4 NKMAX Co., Ltd., Seongnam 13605, Republic of Korea; minchangil@nkmax.com

**Keywords:** natural killer cell, natural killer cell activity, cortisol, dehydroepiandrosterone sulfate, immunity

## Abstract

The adrenal steroid hormones, cortisol and dehydroepiandrosterone sulfate (DHEAS), are associated with the immune system in opposite actions. This study aimed to investigate the relationship between cortisol and DHEAS serum concentrations, their ratio (CDR), and natural killer cell activity (NKA). This cross-sectional study included 2275 subjects without current infection or inflammation in the final analyses. NKA was estimated by measuring the amount of interferon-gamma (IFN-γ) released by activated natural killer cells; low NKA was defined as IFN-γ level < 500 pg/mL. Cortisol, DHEAS levels, and CDRs were categorized by quartiles in men, premenopausal women, and postmenopausal women. Compared with the lowest quartile as reference, the adjusted odd ratios (ORs) and 95% confidence intervals (CIs) for low NKA of the highest cortisol and CDR group were 1.66 (1.09–2.51) and 1.68 (1.11–2.55) in men, 1.58 (1.07–2.33) and 2.33 (1.58–3.46) in premenopausal women, and 2.23 (1.28–3.87) and 1.85 (1.07–3.21) in postmenopausal women. Only in premenopausal women, the highest DHEAS group showed significantly lower risk of low NKA (OR: 0.51, 95% CI: 0.35–0.76). HPA axis activation indicated as high cortisol level, CDR was significantly associated with low NKA, while high DHEAS levels were inversely associated with low NKA in premenopausal women.

## 1. Introduction

Cortisol, an important endogenous glucocorticoid in human, regulates various physiologic processes including metabolism, circadian rhythm, and immunity. Glucocorticoids mediate the flight or fight response to stress, but simultaneously suppress numerous immune functions. On the contrary, the androgenic adrenal steroid hormone, dehydroepiandrosterone sulfate (DHEAS), primarily enhances immunity and has anti-glucocorticoid effects [1]. The cortisol/DHEAS ratio (CDR), a simultaneous evaluation of two steroid hormones with inverse actions, has been recently focused as an informative indicator of catabolic/anabolic balance, chronic stress, and health status [1]. Age-related increase in CDR is considered to be associated with immunological changes observed during ageing [2].

Natural killer (NK) cells are a subset of innate lymphocytes, which protect the host during early immune responses [3]. NK cells rapidly respond to infected or transformed cells by producing lytic molecules such as perforins or granzymes and can produce immune responses through rapid production of various cytokines, interferon-gamma (IFN-γ) being the best characterized among them [4]. Low NK cell activity is related to development of infections and death due to infection in immunologically normal subjects [5,6]. Decreased NK cell activity was seen in various types of tumors, the association being more significant with the advancement of the disease state [7,8,9]. Recent studies reported that patients of coronavirus disease had a significantly decreased number and inhibited function of NK cells [10]. Furthermore, decreased NK cell function in immunologically normal subjects is associated with increased risk of cancer development [11,12,13,14]. Therefore, there is a growing interest in identifying risk factors causing low NK cell activity [15,16,17].

Numerous in vitro studies have investigated the inhibitory effect of glucocorticoids on NK cell function [18,19,20,21,22,23]. Further, a few have shown an inverse relationship between serum cortisol levels and NK cell function in a small number of subjects [24,25,26]. Moreover, several human studies have shown improvements in NK cell numbers and cytotoxicity with DHEA supplementation [27,28]. However, these studies employed complex and time-consuming methods to analyze NK function [29], so the relationship between these steroid hormones and NK function could not be investigated in a large population. Hence, we investigated herein the relationship between cortisol, DHEAS, and their ratio with NK cell activity (NKA) using a simple and novel assay [30,31] in a general large population.

## 2. Materials and Methods

### 2.1. Study Population

**Figure 1:**
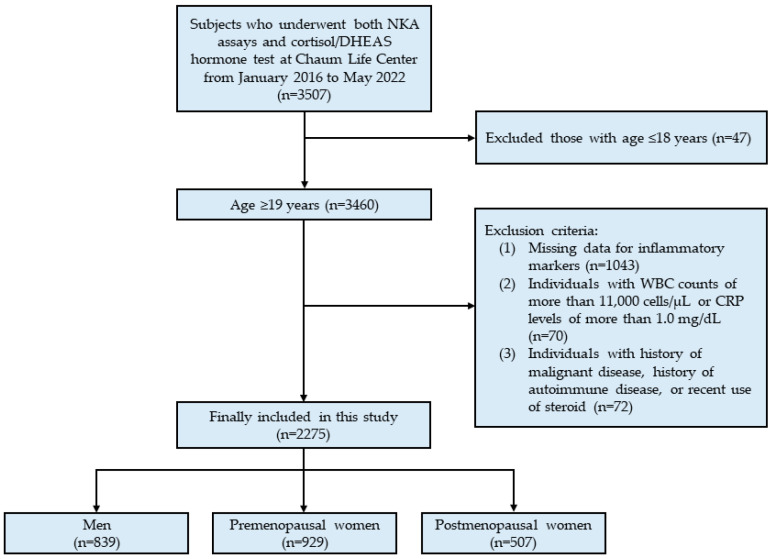
Flowchart of the study population selection.

This cross-sectional study used data from Chaum Life Center. We screened individuals who underwent both NKA assays and cortisol/DHEAS hormone tests at an outpatient clinic or health check-up from January 2016 to May 2022 (*n* = 3507). Data were obtained from the electronic medical records. From 3507 eligible subjects, we included a total of 3460 adults >19 years old. Then, we excluded subjects who met any of the following criteria: (1) missing data for inflammatory markers (*n* = 1043), (2) white blood cell counts >11,000 cells/μL or C-reactive protein levels > 1.0 mg/dL (*n* = 70) due to the possibility of current infection or inflammation [32], (3) history of malignant or autoimmune disease, or recent steroids use (*n* = 72). After exclusion, 2275 subjects (839 men, 929 premenopausal women, and 507 postmenopausal women) were included in the final analysis (Figure 1).

The study protocol was approved by the Institutional Review Board of CHA Bundang Medical Center (CHAMC 2020-10-006). This study was performed in accordance with the principles of the Declaration of Helsinki.

### 2.2. Data Collection

Height (m) and weight (kg) were measured to the nearest 0.1 cm and 0.1 kg, while patients were wearing light-weight clothing and no shoes. Body mass index (BMI) was calculated as the ratio of weight (kg) to height squared (m^2^). Subjects were asked to complete questionnaires about their medical history, including diagnosed diseases and prescribed medications.

Blood samples were collected from the antecubital vein, in the morning between 8 a.m. and 10 a.m. after ≥8 h of fasting. White blood cell (WBC) counts were quantified with an XN-10 Hematology Analyzer (Sysmex, Lincolnshire, IL, USA). Neutrophils and lymphocytes were estimated as proportions of the number of WBCs, and the neutrophil/lymphocyte ratio (NLR) was defined as neutrophil (%) versus lymphocyte (%). C-reactive protein (CRP) was measured with the Hitachi 7600 Analyzer (Hitachi Co., Tokyo, Japan). Cortisol and DHEAS were measured with Cobas 6000 Analyzer (Roche Diagnostics, Rotkreuz, Switzerland). We calculated the cortisol/DHEAS ratio (CDR) as cortisol (μg/dL)/DHEAS (μg/dL) × 100.

### 2.3. IFN-γ Measurement for NKA

NKA in a blood sample was evaluated using a recently developed blood test called NK Vue^®^ Kit, manufactured by NKMAX in Sungnam, Republic of Korea. The test utilized a patented stimulatory cytokine called Promoca^®^ to stimulate IFN-γ secretion in vitro specifically from NK cells in the blood sample, rather than from other immune cells [30,31]. To perform the test, a 1 mL sample of whole blood was collected and transferred into a specialized blood collection tube containing Promoca^®^, and gently mixed within 30 min of collection. The tube was then incubated for 20–24 h in a 37.0 °C chamber to allow for IFN-γ secretion, according to the manufacturer’s instructions. The resulting supernatant was obtained and centrifuged at 3000× *g* for 3 min, and the level of IFN-γ in pg/mL was measured using an enzyme-linked immunosorbent assay (ELISA) plate designed for this purpose. Low NKA was defined as IFN-γ level < 500 pg/mL as in previous studies [12,15].

### 2.4. Statistical Analysis

The normality of the distribution of variables was assessed using the Kolmogorov-Smirnov test. All data are presented as mean ± standard deviation (SD). Differences in clinical characteristics according to sex and menopause, and differences in cortisol, DHEAS, CDR, and NKA values according to age groups, were analyzed using analysis of variance (ANOVA). Cortisol, DHEAS, and CDR were categorized into 4 groups according to quartile values for men, premenopausal and postmenopausal women, respectively, as follows: cortisol (μg/dL), Q1: <6.9, Q2: 6.9–9.1, Q3: 9.1–12.1, Q4: >12.1 for men, Q1: <5.6, Q2: 5.6–7.4, Q3: 7.4–10.2, Q4: >10.2 for premenopausal women, and Q1: <5.6, Q2: 5.6–7.3, Q3: 7.3–9.8, Q4: >9.8 for postmenopausal women; DHEAS (μg/dL), Q1: <129.6, Q2: 129.6–196.3, Q3: 196.3–274.8, Q4: >274.8 for men, Q1: <110.9, Q2: 110.9–157.8, Q3: 157.8–221.5, Q4: >221.5 for premenopausal women, and Q1: <55.5, Q2: 55.5–88.6, Q3: 88.6–129.7, Q4: >129.7 for postmenopausal women; CDR, Q1: <3.0, Q2: 3.0–4.8, Q3: 4.8–7.4, Q4: >7.4 for men, Q1: <3.1, Q2: 3.1–4.6, Q3: 4.6–7.6, Q4: >7.6 for premenopausal women, and Q1: <5.6, Q2: 5.6–8.7, Q3: 8.7–14.3, Q4 >14.3 for postmenopausal women. Differences in NKA levels between the cortisol, DHEAS, and CDR quartiles in each group were analyzed using ANOVA. The proportion of low NKA was calculated according to cortisol, DHEAS, and CDR quartiles in each of the three groups, respectively. The *p* for trend in the ordered quartile groups was estimated using the Cochran–Armitage test. We performed Pearson’s correlation test to calculate the correlation coefficient (r) between cortisol, DHEA, CDR, other inflammatory markers, and NKA. Odds ratios (ORs) and 95% confidence intervals (CIs) for low NKA were calculated using multiple logistic regression analysis after adjusting for WBC count and NLR, which showed a strong correlation with NKA. All statistical analyses were conducted using SPSS statistical software (version 25.0; SPSS Inc., Chicago, IL, USA). A *p*-value <0.05 was considered statistically significant.

## 3. Results

### 3.1. Clinical Characteristics of the Study Population

Table 1 presents the clinical characteristics of the study population. Age, BMI, WBC counts, NLR, CRP, cortisol, DHEAS, CDR, and proportion of participants with hypertension, diabetes mellitus, and dyslipidemia differed between men, premenopausal and postmenopausal women. Only NKA showed no significant difference between groups. Therefore, we analyzed the outcomes by groups.

**Figure 2:**
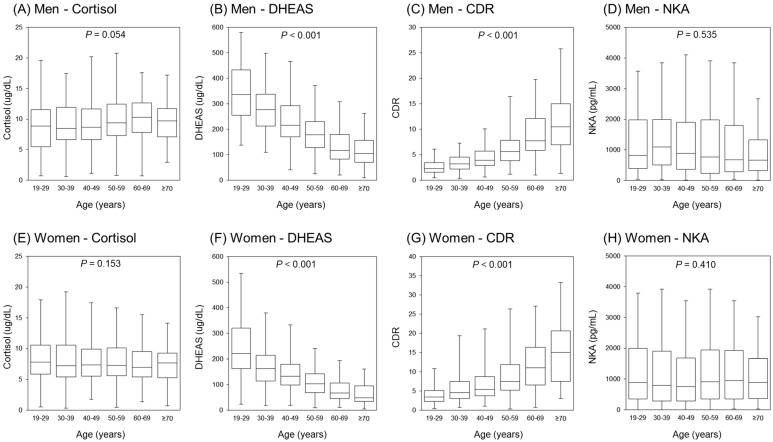
Distribution of cortisol, DHEAS, CDR, and NKA values according to age. Box plots showing the median, interquartile range (IQR), and minimum/maximum data (except for outliers) for each group. *p*-values were calculated using analysis of variance (ANOVA).

The distributions of cortisol, DHEAS, CDR, and NKA values according to age are shown in Figure 2. In both men and women, DHEAS decreased and CDR increased significantly with increasing age (*p* < 0.001), whereas cortisol (*p* = 0.054 in men, and *p* = 0.153 in women) and NKA (*p* = 0.535 in men, and *p* = 0.410 in women) showed no significant differences according to age.

### 3.2. Correlations between Cortisol, DHEAS, CDR, and NKA

**Figure 3:**
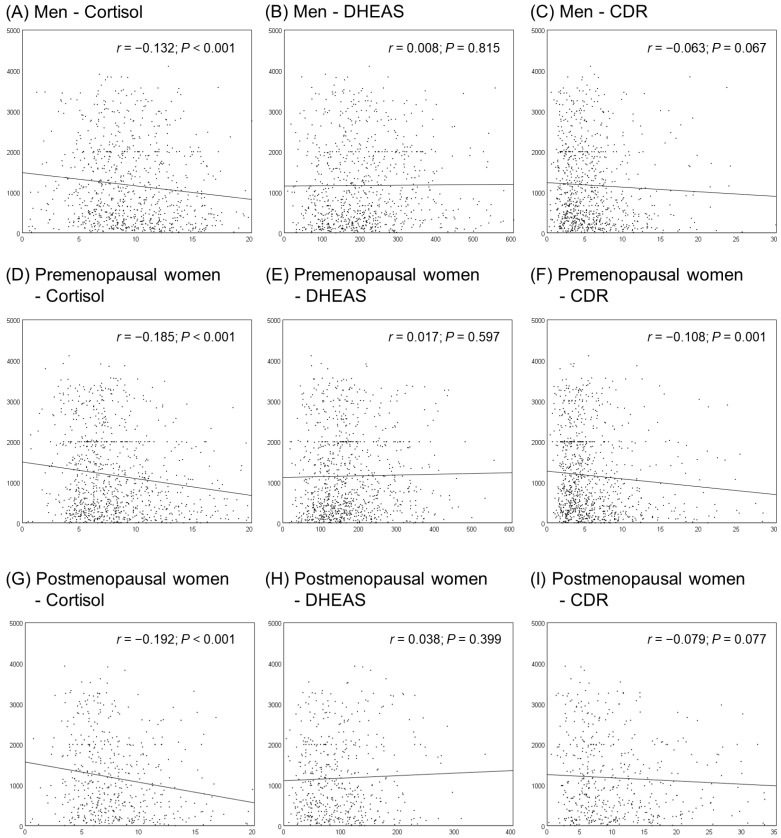
Correlations between cortisol, DHEAS, CDR, and NKA. *r* is correlation coefficient, and *p*-values were calculated using Pearson’s correlation analysis.

The Pearson’s correlation coefficients between cortisol, DHEAS, CDR, and NKA are shown in Figure 3. Cortisol was negatively correlated with NKA in both men and women (*r* = −0.132 in men; *r* = −0.185 in premenopausal women; *r* = −0.192 in postmenopausal women; *p* < 0.001 in all groups), and CDR was negatively correlated with NKA only in premenopausal women (*r* = −0.108; *p* = 0.001).

### 3.3. Comparison of NKA Levels According to Cortisol, DHEAS, and CDR Quartiles

**Figure 4:**
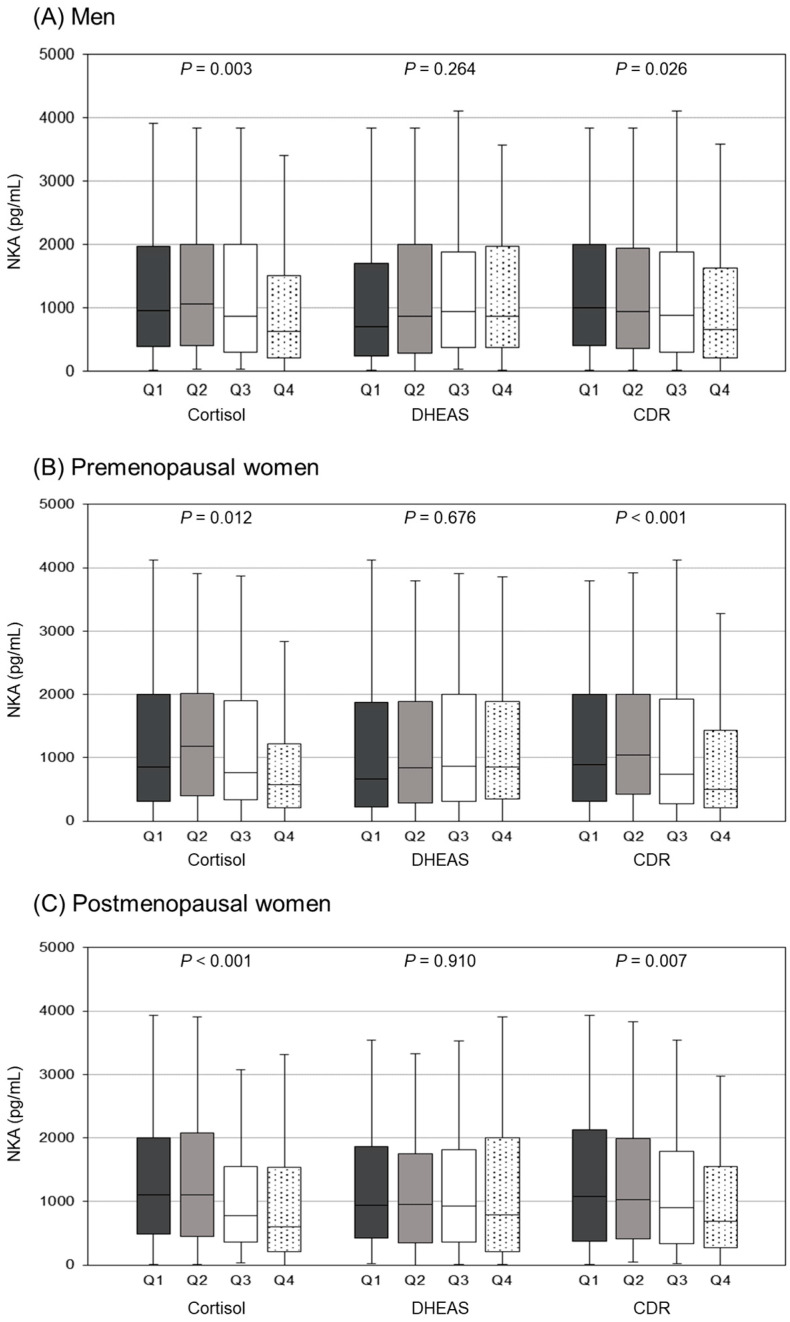
Differences in NKA levels according to group-specific cortisol, DHEAS, and CDR quartiles. Box plots showing the median, interquartile range (IQR), and minimum/maximum data (except for outliers) for each group. *p*-values were calculated using analysis of variance (ANOVA).

Figure 4 shows differences in NKA levels according to cortisol, DHEAS, and CDR quartiles in each group. In both men and women, NKA levels significantly differed between cortisol and CDR quartiles, whereas there were no significant differences between DHEAS quartiles. The cortisol Q4 group showed significantly lower NKA levels than the Q1–3 groups in men and premenopausal women, and the Q1–2 groups in postmenopausal women (*p* < 0.05 by the Tukey’s Honest Significant Difference test; Supplementary Table S1). NKA levels in the CDR Q4 group were significantly lower than the Q1 group in men and postmenopausal women, and lower than the Q1–2 groups in premenopausal women.

### 3.4. Relationship between Cortisol, DHEAS, CDR Quartiles, and Low NKA

**Figure 5:**
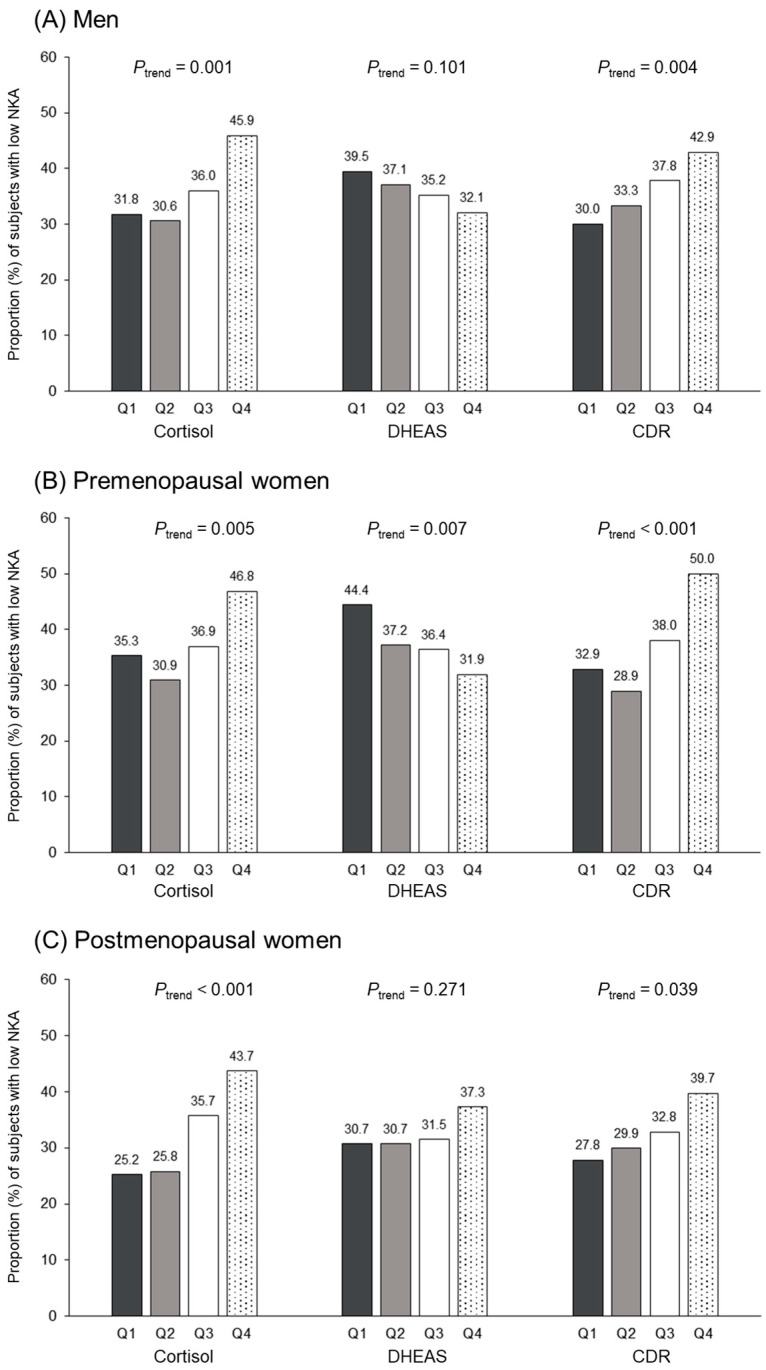
The proportion of subjects with low NKA according to group-specific cortisol, DHEAS, and CDR quartiles. *p* for trend was estimated using Cochran–Armitage test.

Figure 5 shows the proportion of participants with low NKA according to the three group-specific quartiles of cortisol, DHEAS, and CDR levels. The proportion of low NKA increases with increasing cortisol and CDR quartile groups in men, premenopausal and postmenopausal women (*p* for trend < 0.05 by Cochran–Armitage test). Only in premenopausal women, the proportion of low NKA declines with increasing DHEAS quartile groups.

Table 2 represents the results of multiple logistic regression analyses to assess the association between cortisol, DHEAS, CDR quartiles, and low NKA, after adjusting for WBC count and NLR, which showed a significant correlation with NKA levels (Supplementary Table S2). Compared with the lowest cortisol quartile group (Q1) used as reference, the unadjusted ORs (95% CIs) for low NKA of the highest quartile group (Q4) were 1.82 (1.22–2.71) in men, 1.61 (1.11–2.33) in premenopausal women, and 2.30 (1.35–3.92) in postmenopausal women. After adjusting for WBC count and NLR, the adjusted ORs (95% CIs) of the cortisol Q4 group were 1.66 (1.09–2.51) in men, 1.58 (1.07–2.33) in premenopausal women, and 2.23 (1.28–3.87) in postmenopausal women. However, only in premenopausal women, the DHEAS Q3 and Q4 groups showed significantly lower risk of low NKA (OR: 0.64, 95% CI: 0.44–0.94 for Q3, and OR: 0.51, 95% CI: 0.35–0.76 for Q4, respectively) after adjustment. The unadjusted ORs (95% CIs) for low NKA of the highest CDR quartile group (Q4) were 1.75 (1.17–2.62) in men, 2.04 (1.40–2.97) in premenopausal women, and 1.71 (1.01–2.90) in postmenopausal women, compared with the lowest CDR quartile group (Q1). After adjustment for WBC counts and NLR, the CDR Q4 group showed significantly higher risk of low NKA (OR: 1.68, 95% CI: 1.11–2.55 for men, OR: 2.33, 95% CI: 1.58–3.46 for premenopausal women, and OR: 1.85, 95% CI: 1.07–3.21 for postmenopausal women, respectively).

## 4. Discussion

The present study showed that high serum cortisol levels and CDR were significantly associated with low NKA in both men and women, while high DHEAS was inversely associated with low NKA only in premenopausal women. In addition, we found that DHEAS decreased and CDR increased with age, but there were no significant differences in cortisol and NKA with age in both men and women.

Our study showed similar results to previous studies in that DHEAS decreased and CDR increased significantly with age in both men and women. Immunosenescence refers to a deterioration of immune response associated with aging [33]. Mainly characterized by defective T cell response, there are changes in other cells of the innate immune system. A previous study reported a clear age-related decline of DHEAS secretion, no age-related significant difference in morning serum cortisol levels, and a significant age-related increase in CDR [34]. However, studies on aging and NK cells have shown inconsistent results. Several studies have found an increase in cells with high NK activity with increasing age, suggesting NK cell activity does not heavily deteriorate with age [35]. On the other hand, other studies have suggested a progressive increase in the percentage of NK cells with a mature phenotype and impaired cytotoxic capacity when considered on an “per cell” basis with aging [36,37]. In our study, NKA showed no significant differences according to age in both men and women.

Similar to previous studies [15,38], NKA was negatively correlated with WBC count and NLR in our study. This inverse correlation suggests that NKA could be a clinically significant index reflecting immune function and inflammatory status. Accordingly, WBC count and NLR were adjusted in multivariate models to reduce the effects of WBC count and NLR on low NKA. However, these potential confounders did not influence the relationship between adrenal hormones and NKA.

We found that high cortisol and high CDR in both men and women were significantly associated with low NKA, after adjusting for inflammatory markers—WBC count and NLR. The possible mechanisms of the interaction between high cortisol and low NKA are based on several direct effects of glucocorticoids on the immune system. Previous studies have reported the inhibitory effect of glucocorticoids on NK function [18,19,20,21,22,39,40,41,42]. After binding to the glucocorticoid receptor, glucocorticoids alter gene transcription, reducing the expression of genes important for NK function [21,41], like integrin LFA-1, which allows NK cells to adhere to target cells, or effector molecules such as perforin, granzyme A and B, which reduce the cytotoxicity of NK cells [20,22]. The surface expression of the NK cytotoxicity receptors, such as NKp64 and NKp30, are down-regulated by glucocorticoids [43]. Glucocorticoids are also possible peripheral clock synchronizers, which have been demonstrated to affect molecular clock genes expression, such as PER1-3 and BMAL1, in human peripheral cells including NK cells [44,45]. In 2017, Bancos et al. [46] showed for the first time a significantly decreased NK cell cytotoxicity in patients with primary adrenal insufficiency even after hydrocortisone and DHEA replacement, suggesting that the lack of diurnal cortisol delivery altered peripheral clock gene regulation of immune cells. Moreover, IFN-γ production is inhibited by glucocorticoids [47,48]. The inverse relationship between cortisol and NKA has been reported in several human studies [24,25,26]. However, because of the methodological complexity of measuring NK cell function, these studies analyzed a small number of subjects under experimental environments. In one study, although NK cell count was positively associated with cortisol levels, the relationship between cortisol levels and NK cell function was negative, similar to our results [25]. This may suggest that the progressive increase in the percentage of NK cells with aging [36,37] is not associated with NK cell function.

High DHEAS was inversely associated with low NKA only in premenopausal women and not in postmenopausal women or men. Although the precise mechanism by which high DHEAS significantly lowered the risk of low NKA in premenopausal women is unclear, differences in sex hormones of premenopause and postmenopause might have contributed to this discrepancy. Estradiol has immune-enhancing functions [49], and as such, the incidence of chronic inflammatory disorders in women increases after menopause [50]. Thus, DHEA might effectively influence immunity in an estrogen-rich environment. Since the changes in immune responses after menopause are not yet clearly elucidated, more studies are needed in the future.

This study has several limitations. First, it was conducted in a single center, which hinders in generalizing the results. However, our study included a relatively large sample. Second, NKA was only measured using an IFN-γ assay. Although NK cell count was not measured, this new assay is advantageous for its simplicity, which makes it more appropriate for large-scale studies. Third, cortisol was measured a single time; since it follows a diurnal pattern, this might have some limitations. However, our study included a relatively large sample and cortisol was measured simultaneously with NKA. Fourth, medical conditions that could affect serum cortisol, DHEAS, or NKA levels, such as postmenopausal hormone therapy or supplementations, were not evaluated. Finally, the causal relationship between high cortisol, high CDR, and low NKA could not be demonstrated because of the cross-sectional study design. Further longitudinal studies are required to determine the causal relationship between adrenal hormones and immunity. Despite these weaknesses, our study has the strength of investigating whether serum cortisol, DHEAS, and their ratio correlates with NK cell function measured using a relatively simple novel assay in a large population of subjects.

## 5. Conclusions

In the general population in our study, serum cortisol levels were associated with low NKA, whereas DHEAS levels were inversely associated with low NKA only in premenopausal women. Our results suggest that two adrenal steroid hormones may modulate NK cell function with opposite actions. Further prospective studies are needed to demonstrate the causal relationship underlying this relationship and the precise mechanisms of sex and menopausal differences at play.

## Figures and Tables

**Table 1:** Clinical characteristics of the study population.

	Men	Premenopausal Women	Postmenopausal Women	*p*-Value
N	839	929	507	
Age (years)	49.9 ± 12.8	36.9 ± 1.5	58.6 ± 7.6	<0.001
BMI (kg/m^2^)	25.6 ± 4.0	21.3 ± 3.0	23.0 ± 3.5	<0.001
WBC count (cells/μL)	5.80 ± 1.51	5.61 ± 1.51	5.31 ± 1.40	<0.001
NLR	1.69 ± 0.77	1.84 ± 0.89	1.55 ± 0.70	<0.001
CRP (mg/dL)	0.13 ± 0.15	0.11 ± 0.15	0.14 ± 0.17	<0.001
Cortisol (μg/dL)	9.7 ± 4.0	8.5 ± 4.6	8.0 ± 3.7	<0.001
DHEAS (μg/dL)	213.9 ± 115.4	175.8 ± 91.7	99.0 ± 58.2	<0.001
CDR	6.2 ± 5.6	6.3 ± 5.7	11.3 ± 9.6	<0.001
NKA (pg/mL)	1168.2 ± 997.6	1154.6 ± 1009.4	1168.7 ± 960.9	0.948
Hypertension, *n* (%)	125 (14.9)	11 (1.2)	101 (19.9)	<0.001
Diabetes mellitus, *n* (%)	55 (6.5)	2 (0.2)	35 (7.0)	<0.001
Dyslipidemia, *n* (%)	148 (17.6)	20 (2.1)	138 (27.3)	<0.001

Data are expressed as mean ± SD. *p*-values were calculated using analysis of variance (ANOVA). Abbreviations: BMI, body mass index; CDR, cortisol/DHEAS ratio; CRP, C-reactive protein; DHEAS, dehydroepiandrosterone sulfate; NKA, natural killer cell activity; NLR, neutrophil/lymphocyte ratio; WBC, white blood cell.

**Table 2:** Odds ratios and 95% confidence intervals for low NKA by cortisol, DHEAS, and CDR quartiles in each sex.

	Cortisol Quartiles			
	Q1	Q2	Q3	Q4
Men				
Unadjusted	1.00 (Reference)	0.95 (0.63–1.43)	1.21 (0.81–1.81)	1.82 (1.22–2.71)
Adjusted	1.00 (Reference)	0.95 (0.62–1.45)	1.21 (0.80–1.84)	1.66 (1.09–2.51)
Premenopausal women				
Unadjusted	1.00 (Reference)	0.82 (0.56–1.20)	1.07 (0.73–1.56)	1.61 (1.11–2.33)
Adjusted	1.00 (Reference)	0.85 (0.57–1.27)	1.03 (0.70–1.52)	1.58 (1.07–2.33)
Postmenopausal women				
Unadjusted	1.00 (Reference)	1.03 (0.59–1.81)	1.65 (0.96–2.84)	2.30 (1.35– 3.92)
Adjusted	1.00 (Reference)	1.01 (0.56–1.82)	1.62 (0.92–2.82)	2.23 (1.28–3.87)
	DHEAS quartiles			
	Q1	Q2	Q3	Q4
Men				
Unadjusted	1.00 (Reference)	0.90 (0.61–1.34)	0.83 (0.56–1.24)	0.72 (0.48–1.08)
Adjusted	1.00 (Reference)	0.97 (0.64–1.45)	0.93 (0.62–1.39)	0.68 (0.45–1.03)
Premenopausal women				
Unadjusted	1.00 (Reference)	0.74 (0.51–1.07)	0.72 (0.49–1.04)	0.59 (0.40–0.86)
Adjusted	1.00 (Reference)	0.74 (0.51–1.08)	0.64 (0.44–0.94)	0.51 (0.35–0.76)
Postmenopausal women				
Unadjusted	1.00 (Reference)	1.00 (0.59–1.70)	1.04 (0.61–1.77)	1.34 (0.80–2.26)
Adjusted	1.00 (Reference)	1.09 (0.63–1.88)	1.03 (0.59–1.77)	1.26 (0.73–2.16)
	CDR quartiles			
	Q1	Q2	Q3	Q4
Men				
Unadjusted	1.00 (Reference)	1.17 (0.78–1.76)	1.42 (0.94–2.13)	1.75 (1.17–2.62)
Adjusted	1.00 (Reference)	1.22 (0.80–1.87)	1.47 (0.96–2.23)	1.68 (1.11–2.55)
Premenopausal women				
Unadjusted	1.00 (Reference)	0.83 (0.56–1.23)	1.25 (0.86–1.83)	2.04 (1.40–2.97)
Adjusted	1.00 (Reference)	0.92 (0.62–1.38)	1.33 (0.89–1.96)	2.33 (1.58–3.46)
Postmenopausal women				
Unadjusted	1.00 (Reference)	1.11 (0.64–1.91)	1.27 (0.74–2.17)	1.71 (1.01–2.90)
Adjusted	1.00 (Reference)	1.15 (0.65–2.02)	1.36 (0.78–2.38)	1.85 (1.07–3.21)

Odds ratios were calculated after adjustment for WBC count and NLR. Abbreviations: CDR, cortisol/DHEAS ratio; DHEAS, dehydroepiandrosterone sulfate; NKA, natural killer cell activity.

## Data Availability

All datasets used and/or analyzed during the study are available from the corresponding author upon reasonable request.

## Supplementary Materials

The following supporting information can be downloaded at: https://www.mdpi.com/article/10.3390/jcm12124027/s1, Table S1: Differences in NKA levels according to cortisol, DHEAS, and CDR quartiles; Table S2: Correlation coefficients (*r*) between NKA and other variables including inflammatory markers.
