# Supplementary material for: Relationship between Serum Cortisol, Dehydroepiandrosterone Sulfate (DHEAS) Levels, and Natural Killer Cell Activity: A Cross-Sectional Study
Source: J Clin Med. 2023 Jun 13;12(12):4027. doi: 10.3390/jcm12124027 (PMC10299486; doi:10.3390/jcm12124027)
Supplement: Supplementary file 1 [file jcm-12-04027-s001.zip › jcm-2306421-supplementary.pdf]

Table S1. Differences in NKA levels according to cortisol, DHEAS, and CDR quartiles

Men			Premenopausal women			Postmenopausal women		
	NKA (pg/mL)	*p*		NKA (pg/mL)	*p*		NKA (pg/mL)	*p*
Cortisol		0.003	Cortisol		<0.001	Cortisol		<0.001
Q1 (≤ 6.9)	1233.5 ± 998.7[d]		Q1 (≤ 5.6)	1269.4 ± 1081.1[d]		Q1 (≤ 5.6)	1332.8 ± 1017.4[d]	
Q2 (7.0-9.1)	1280.4 ± 1017.0[d]		Q2 (5.7-7.4)	1351.3 ± 1026.0[d]		Q2 (5.7-7.3)	1377.8 ± 1032.8[c,d]	
Q3 (9.2-12.1)	1203.1 ± 1019.7[d]		Q3 (7.5-10.2)	1147.1 ± 1029.2[d]		Q3 (7.4-9.8)	1029.5 ± 845.2[b]	
Q4 (≥ 12.2)	950.3 ± 924.5[a,b,c]		Q4 (≥ 10.3)	848.4 ± 812.3[a,b,c]		Q4 (≥ 9.9)	930.0 ± 864.3[a,b]	
DHEAS		0.264	DHEAS		0.676	DHEAS		0.910
Q1 (≤ 129.6)	1054.3 ± 969.6		Q1 (≤ 110.9)	1120.2 ± 1078.9		Q1 (≤ 55.5)	1178.3 ± 919.9	
Q2 (129.7-196.3)	1222.7 ± 1070.1		Q2 (111.0-157.8)	1122.5 ± 976.5		Q2 (55.6-88.6)	1131.8 ± 902.3	
Q3 (196.4-274.8)	1222.0 ± 1019.8		Q3 (157.9-221.5)	1221.8 ± 1032.7		Q3 (88.7-129.7)	1149.9 ± 920.0	
Q4 (≥ 274.9)	1173.8 ± 922.0		Q4 (≥ 221.6)	1154.3 ± 947.9		Q4 (≥ 129.8)	1215.0 ± 1098.6	
CDR		0.026	CDR		<0.001	CDR		0.007
Q1 (≤ 3.0)	1277.1 ± 992.5[d]		Q1 (≤ 3.1)	1285.2 ± 1047.4[d]		Q1 (≤ 5.6)	1363.2 ± 1088.6[d]	
Q2 (3.1-4.8)	1217.8 ± 1005.1		Q2 (3.2-4.6)	1269.8 ± 987.6[d]		Q2 (5.7-8.7)	1217.8 ± 929.7	

Q3 (4.9-7.4)	1180.8 ± 1021.5	Q3 (4.7-7.6)	1145.9 ± 1028.2	Q3 (8.8-14.3)	1145.1 ± 974.3
Q4 (≥ 7.5)	997.2 ± 955.2[a]	Q4 (≥ 7.7)	918.0 ± 934.1[a,b]	Q4 (≥ 14.4)	948.6 ± 791.7[a]

Data are expressed as mean ± SD. *p*-values were calculated using analysis of variance (ANOVA).
[a] *p*-value <0.05 vs. Q1 group based on the Tukey's Honest Significant Difference test.
[b] *p*-value <0.05 vs. Q2 group based on the Tukey's Honest Significant Difference test.
[c] *p*-value <0.05 vs. Q3 group based on the Tukey's Honest Significant Difference test.
[d] *p*-value <0.05 vs. Q4 group based on the Tukey's Honest Significant Difference test.
Abbreviations: CDR, cortisol/DHEAS ratio; DHEAS, dehydroepiandrosterone sulfate; NKA, natural killer cell activity.

Table S2. Correlation coefficients (*r*) between NKA and other variables including cortisol, DHEAS, and CDR

	Men		Premenopausal women		Postmenopausal women	
	r	*p*	*r*	*p*	*r*	*p*
Age	-0.055	0.110	-0.057	0.084	-0.054	0.228
WBC count	-0.174	<0.001	-0.189	<0.001	-0.142	0.001
NLR	-0.284	<0.001	-0.209	<0.001	-0.302	<0.001
CRP	-0.035	0.332	-0.076	0.035	-0.095	0.043
Cortisol	-0.132	<0.001	-0.185	<0.001	-0.192	<0.001
DHEAS	0.008	0.815	0.017	0.597	0.038	0.399
CDR	-0.063	0.067	-0.108	0.001	-0.079	0.077

r is correlation coefficient, and *p*-values were calculated using Pearson's correlation analysis.
Abbreviations: CDR, cortisol/DHEAS ratio; CRP, C-reactive protein; DHEAS, dehydroepiandrosterone sulfate; NLR, neutrophil/lymphocyte ratio; WBC, white blood cell.
